# Genes and physiological strategies in bacterial antibiotic resistance

**DOI:** 10.3389/fmicb.2026.1811959

**Published:** 2026-05-08

**Authors:** Muhammad Shahid Nadeem, Rabia Rasool, Muhammad Afzal, Inam Ullah, Bibi Nazia Murtaza, Rabeea Mustafa Ali Daoub, Kamel Chaieb, Sami I. Alzarea, Imran Kazmi, Ghulam Md Ashraf

**Affiliations:** 1 Department of Biochemistry, Faculty of Science, King Abdulaziz University, Jeddah, Saudi Arabia; 2 Institute of Molecular Biology and Biotechnology, The University of Lahore, Lahore, Pakistan; 3 Department of Pharmaceutical Sciences, Pharmacy Program, Batterjee Medical College, Jeddah, Saudi Arabia; 4 Department of Zoology, Faculty of Science, Abbottabad University of Science and Technology (AUST), Abbottabad, Pakistan; 5 Department of Chemistry, College of Science, Northern Border University, Arar, Saudi Arabia; 6 Department of Pharmacology, College of Pharmacy, Jouf University, Sakaka, Al Jouf, Saudi Arabia; 7 Department of Biomedical Sciences, College of Medicine, Gulf Medical University, Ajman, United Arab Emirates

**Keywords:** bacterial drug resistance, genes, genetics, mechanisms, physiology

## Abstract

Bacteria display an incredible genetic plasticity, which enables them to adapt to various environmental stressors, such as antibiotic compounds that may imperil their existence. Bacterial resistance mechanisms include degradative enzymes, inactivation of antibiotics, antibacterial target site mutation, change in target, altered cell wall permeability to antibiotics, bypass of metabolic pathways, and efflux pumping of antibiotics across the cell membrane. These mechanisms are encoded by genomic changes ranging from point mutation via genetic elements assembly to horizontal transfer of genes from the environment. Antibiotic resistance in bacteria can be inherited or acquired. Antibacterial resistance genes may accumulate mobile elements, leading to multi-drug-resistant phenotypic transfer via a single genetic event. The resistance to antibiotics has been frequently increasing in clinical settings, which drives scientists to research alternative antibacterial medicines to prevent the growth and spread of drug-resistant bacteria. Technological advancements and the discovery of innovative drug moieties with targeting potential have led to the development of novel drug compounds with diverse therapeutic properties. This includes alternative cellular, physiological, and metabolic patterns of bacteria that may be potential pharmacological targets for the next generation of antibiotics. It is beneficial to characterize antibiotic resistance genotypes and phenotypes causing antibiotic bacterial resistance. An understanding of mechanisms that lead to the development and spread of antibiotic resistance will help clinicians in making appropriate decisions regarding antibiotic usage in a wide range of circumstances. The current review has highlighted the mechanism of drug resistance in bacteria, and has enlisted the antibiotic resistance genes (ARGs) and their importance in aggravating the resistance phenomenon.

## 1 Introduction

Antibiotics are the bioactive compounds, produced by microorganisms or chemically synthesized, having the capability to kill or hinder the growth of bacteria (Serna-Galvis et al., 2020). The golden era of antibiotics started with the commercial-scale production of streptomycin and penicillin, which transfigured the treatment of infectious diseases and significantly reduced morbidity and mortality worldwide (Hutchings et al., 2019). Since then, antibiotics have been extensively used for the prevention and treatment of bacterial infections in modern medicine, in some cases, protozoan infections. The prescription of antibiotics mainly depends on the pathogen identification; if the pathogen is unknown, empirical therapy via broad-spectrum antibiotics is commonly adopted based on signs and symptoms. However, if the disease-causing pathogen is identified, the therapy with narrow-spectrum antibiotics is preferred to increase efficacy and minimize resistance development (Sullivan, 2022; Massele et al., 2023). Antibiotics can be administered through various routes, such as oral, intravenous, or topical applications, depending on the nature and severity of infection (Long et al., 2024; Glasziou et al., 2022). Despite their therapeutic benefits, various adverse effects are associated with antibiotics depending on the type of antibiotic class, the targeted microbes, and the overall health status of patients. These effects may encompass pharmacological, toxicological reactions, as well as hypersensitivity and allergic reactions (Menon et al., 2018). Based on their chemical structure, mode of action, and antimicrobial spectrum, antibiotics are classified into several major groups; penicillins (inhibitors of cell wall synthesis), aminoglycosides (bacterial protein synthesis inhibitors that target 30S subunit of ribosomes), cephalosporins (cell wall synthesis inhibitors), quinolones (inhibitors of bacterial type II topoisomerase halting the DNA transcription and replication), monobactams (cell wall inhibitors in aerobic Gram negative bacteria), carbapenems (broad spectrum cell wall synthesis inhibitors), macrolides (reversibly bind to the 50S subunit of ribosomes and inhibit the bacterial protein synthesis), sulphonamides (inhibitors of folic acid synthesis that disrupt the bacterial growth), chloramphenicol (bacterial protein synthesis inhibitors), oxazolidinones (reversibly bind to 50S subunit and inhibit the 70S formation to stop bacterial protein synthesis), and tetracyclines (inhibit the binding of aminoacyl-tRNA to the ribosome-mRNA complex and inhibit the protein synthesis) (Schuetz et al., 2025; Su et al., 2025). The antibacterial activity of antibiotics is based on their highly specific interactions with distinct sites on cellular or extracellular components of a given target organism. These targets are usually critical components of bacterial survival and replication, identified through genomic and biochemical studies. Broadly, antibiotic drugs act via either blocking the major pathways or by interfering with the cellular structures. The most widely used antibiotics can be grouped into four principal categories: the cell wall or cell membrane synthesis inhibitors, protein synthesis inhibitors, nucleic acid (DNA or RNA) synthesis inhibitors, and inhibitors of folate pathways (Ma et al., 2026; Wilson et al., 2020). The bacterial cell wall, primarily made of peptidoglycan, is the major target for several antibiotics (Butler and Capon, 2026). The beta-lactam antibiotics mainly inhibit bacterial cell wall synthesis by binding to penicillin-binding proteins (PBP), thereby disrupting the peptidoglycan cross-linking and cell division (Schaenzer and Wright, 2020; Ene et al., 2026). Another class of antibiotics, glycopeptides, inhibits cell wall synthesis by binding to peptidal d-alanyl-d-alanine termini of precursor peptidoglycan subunits and impede their binding with PBPs (Amin-Chowdhury et al., 2026). Protein synthesis inhibitors, such as aminoglycosides, tetracyclines, chloramphenicol, macrolides, and oxazolidinones, inhibit protein synthesis by targeting the bacterial ribosomal subunits and interfere with translation. Similarly, quinolones and fluoroquinolones inhibit bacterial DNA replication by targeting DNA gyrase and topoisomerase IV, the enzyme essential for DNA double-strand supercoiling and segregation during cell division (Sullivan et al., 2020; Osterman et al., 2020). Folate pathway inhibitors, sulfonamides, competitively inhibit dihydropteroate synthase with a higher infinity than natural substrate, *p*-amino benzoic acid, while trimethoprim inhibits the enzyme dihydrofolate reductase involved in the last stages of folic acid metabolism (Verma et al., 2022; Mondal and Malakar, 2020). The pathogenic bacteria are specifically adapted and endowed to overcome the host immune barrier and colonize various body sites. Some pathogenic bacteria are restricted to epithelial surfaces of the body, while many others invade deeply in blood and tissues, disseminating lymphatic and blood streams, leading to systemic infections (Li et al., 2021). This adaptive capacity is closely interconnected to genetic architecture of bacteria, particularly the presence and dissemination of antibiotic resistance genes (ARGs), which translate molecular mechanisms that directly affect resistance phenotypes.

Antibiotic resistance (AR), with an estimated 4.95 million deaths annually linked to resistant infection and projections reaching 10 million deaths per year by 2050, is recognized as one of the most critical global health threats of the 21st century (Lessa and Sievert, 2023). Resistant rates are high among key pathogens, such as ~42% in *Escherichia coli* to third-generation cephalosporin, sans ~35% methicillin resistance in *Staphylococcus aureus*. The burden is particularly severe in low- and middle-income countries due to antibiotic misuse, limited surveillance, and healthcare constraints (Alomari et al., 2024; World Health Organization, 2024). Importantly, AR is not a modern phenomenon but an ancient evolutionary adaptation, as resistance genes have been identified in environmental and permafrost microbiomes predating clinical antibiotic use (Saleem et al., 2024; Haan and Drown, 2021). However, its rapid escalation into a global crisis has been largely driven by anthropogenic factors, such as antibiotic overuse, agricultural practices, inadequate infection control, and global dissemination of resistant strains. At the molecular level, this global challenge is underpinned by bacterial genetic plasticity, a primary determinant of the physiology of bacteria that enables rapid adaptation to diverse and often harsh environments. This plasticity arises through mutation-based adaptation, horizontal gene transfer (HGT), and regulatory gene networks modulation controlling pathways associated with resistance. Spontaneous mutations can change the regulatory elements or targets, declining drug susceptibility, while HGT enhances ARGs acquisition and spread across the dynamic bacterial population. These determinants are expressed as coordinated physiological adaptation by strictly regulating gene expression networks, such as increased efflux activity, diminishing membrane permeability, activation of stress response, and metabolic reprogramming, which ultimately contribute to phenotypic resistance. Hence, mechanisms involved in physiological resistance should be interpreted as gene alteration functional outputs rather than as an independent process. These integrated physiological and genetic mechanisms promote the survival of bacteria under antibiotic pressure. Recent advances in metagenomics and systems biology further elaborate that rather than single-gene effects, resistance arises from interlinked gene regulatory networks, supporting the genotypic-to-phenotypic continuum of antibiotic resistance (Mehra and Hintze, 2024).

In this context, this review aims to provide a comprehensive overview of recent developments in antibiotic resistance. It also discusses the interaction between genetic determinants, such as antibiotic resistance genes and regulatory elements, and their corresponding physiological expressions, such as multidrug resistance, genes and genetic factors involved, and physiological strategies associated with drug resistance.

## 2 Antibiotic resistance and multidrug resistance

Antibiotic resistance started back in the 1950s when resistance against penicillin was reported (Mirzakarimovna, 2026). The problem was temporarily resolved by the introduction of beta-lactam antibiotics. Again, the first case of methicillin-resistant *Staphylococcus aureus* (MRSA) was reported in 1961 (Amin-Chowdhury et al., 2026; Mirzakarimovna, 2026; Adrizain et al., 2018). The next two decades were indeed the golden era for the discovery of novel classes of antibiotics, which brought revolution in therapies against infectious diseases (Baloch et al., 2020). With more than 2 million annual infections and 700 deaths, bacterial resistance is one of the biggest threats to human health in the 21st century (Chokshi et al., 2019). About 20,000 bacterial genes associated with drug resistance have been identified so far (Mirzakarimovna, 2026; Teng et al., 2026). In the recent past, an ever-increasing bacterial resistance and decline in the development of new antibiotics have made infections like pneumonia and tuberculosis harder to treat (Sheam et al., 2020; Khawbung et al., 2021).

### 2.1 Mechanisms of antibiotic resistance

Bacteria have evolved various mechanisms to counter the action of inhibitory antibiotics (Wilson et al., 2020). The bacteria are referred to as resistant bacteria if they continue to grow and replicate in the presence of antibiotics (Darby et al., 2023; Bonev and Brown, 2019). The detailed understanding of cellular and molecular mechanisms behind this phenomenon aids in the development of novel therapeutic approaches and strategies that might block or evade this resistance. As befits a mature study field, the beginning of which predates the clinical penicillin development, by now, gathered extensive knowledge regarding the nature of resistance in different classes of antibiotics, such as drug efflux and drug degradation, as well as the mutation and modification of the target (Laxminarayan and Brown, 2001). Several molecular mechanisms mediate antibiotic resistance. For instance, enzymatic degradation involves enzyme production, such as beta-lactamases in *Staphylococcus aureus*, which inactivate the antibiotic by hydrolyzing the β-lactam ring (Nusrat et al., 2025). Efflux pump systems, such as AcrAB-TolC in *E. coli*, reduce intracellular concentration of drug below therapeutic levels via actively transporting antibiotics out of the cell (Obaid et al., 2026). Modification of the target via gene mutation encoding antibiotic targets occurs; for example, a decline in fluoroquinolone binding affinity through mutation in the gyrA gene alters the DNA gyrase structure (Elbaiomy et al., 2025). Moreover, reduced membrane permeability results from alteration or loss of porin proteins restricting antibiotic entry into the cell, particularly in Gram-negative bacteria. These mechanisms often act synergistically together, increasing the survival of bacteria under pressure (Misra, 2026; Sartori et al., 2026). In clinical settings, resistance mechanisms rarely act alone. For instance, carbapenem-resistant *Klebsiella pneumoniae* typically exhibits a combination of beta-lactamase production, such as KPC enzymes, which decrease membrane permeability and enhance efflux activity, all of which contribute to compromised treatment outcomes and higher mortality. Similarly, multidrug-resistant *Pseudomonas aeruginosa* relies on multiple coordinated strategies, such as efflux pump overexpression and biofilm formation, which greatly restrict options for treatment and prolong hospitalization (Naylor et al., 2025).

### 2.2 Genetic basis of resistance development

As described above, bacteria seem to have a natural mechanism that promotes resistance. It arises via mutations at the DNA level (McKenna, 2020). However, bacteria possess the ability to transfer genetic material among themselves directly via plasmids, demonstrating the fact that natural selection is not the sole mechanism for developing resistance. Bacterial colonies also mutate, developing resistance (Alenazi et al., 2020). Another reason for the development of resistance is the incomplete course of prescribed antibiotics, as bacteria remain unaffected and are more immune to the antibiotic actions (Mubeen et al., 2021). Bacterial antibiotic resistance can develop through various mechanisms, such as enzymic inactivation of the antibiotic to make it ineffective against bacteria or drug excretion via efflux pumps, in which bacteria fasten antibiotic elimination by stimulating various proteins that in turn remove extensive substances from the periplasm outside the cell. Resistance can also develop through the reduction in absorption of a substance across the cell membrane due to an alteration in its permeability. Drug target modification, demolishing the binding efficacy of antibiotics and reducing their potential can also be the reason of the resistance (McArthur, 2019). Another reason behind resistance development is the unnecessary and inappropriate antibiotic usage (Kojima, 2019; van Duin and Paterson, 2020). Additionally, mutation rates, under antibiotic pressure, and HGT of resistance determinants through plasmids, transposons, and integrons strongly influence resistance development. The mechanisms of HGT, such as transformation, conjugation, and transduction, facilitate the rapid dissemination of ARGs across the bacterial population (Wachino, 2025).

### 2.3 Types of resistance

**Figure 1:**
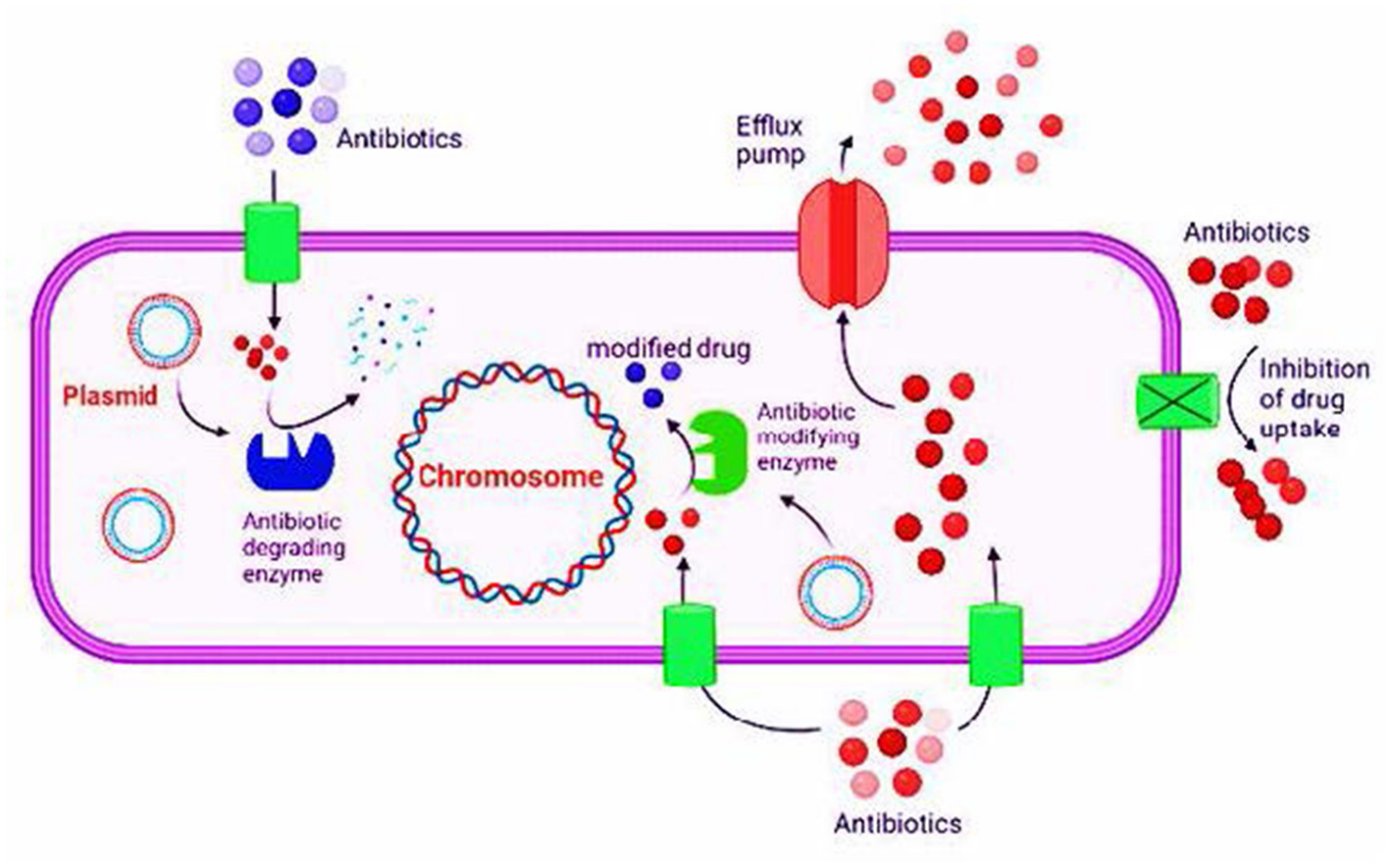
An overview of antibiotic resistance mechanisms in bacteria.

The resistance to antibiotics is categorized into four types: natural resistance, acquired resistance, cross-resistance, and multiple-drug resistance. Natural resistance is caused by bacterial structural characteristics but is not associated with antibiotic usage. It is not linked to hereditary property and develops as a result of structural modification, as the bacteria lack the structure of the targeted antibiotic, or the antibiotic does not reach its target due to its characteristics. For instance, Gram-negative bacteria are resistant to vancomycin because it cannot cross the outer membrane of bacteria. Similarly, mycoplasma and urea plasma, absent from the cell wall, are naturally resistant to beta-lactams (Sprong et al., 2020; Baquero et al., 2021). Acquired resistance develops as a result of modifications in bacterial genetic characteristics. It may occur to antibiotics once it has been responsive before. Acquired resistance may occur due to structures of the chromosome or extra-chromosomal entities, such as plasmids and transposons (Zhu et al., 2022). Chromosomal mutation may occur due to physical and chemical factors that lead to structural changes in the bacterial cell, which in turn, reduce the permeability of the bacterial drug or may alter the drug targets. Streptomycin, erythromycin, lincomycin, and aminoglycosides encounter this type of resistance (Naveed et al., 2020; Gajic et al., 2025). Extra chromosomal resistance is linked to plasmids, transposons, and integrons (Bereznyakov et al., 2022). Cross-resistance is developed in bacteria that are resistant to certain drugs and also become resistant to other drugs with the same structure or mechanism. Erythromycin and neomycin are vulnerable to such resistance (Kreft and Wohlrab, 2022; Ramsey and Trubiano, 2025). For example, intrinsic resistance is seen in Gram-negative bacteria against vancomycin, cross-resistance is often observed among macrolide antibiotics due to shared targets, and acquired resistance is illustrated by MRSA through the mecA gene (Harris et al., 2023). Cephalosporin and penicillin are also evident with cross-resistance, which may have chromosomal or extra chromosomal origin (Wang J. et al., 2017) (Figure 1).

### 2.4 Clinical and epidemiological impact of MDR, XDR, and PDR

Multi-drug resistant (MDR—a condition where the bacteria acquire resistance against at least one member agent from three or more antibiotic groups) bacteria become resistant to the antibiotics that are used to treat them. The inappropriate use of antibiotic therapy might result in such kind of resistance. MDR in bacteria can occur via two mechanisms: either by accumulating multiple genes, each coding for resistance to a particular drug, or increased expression of genes that code for multidrug efflux pumps, inactivation of enzymes, or modification in target structures (Akram et al., 2020). If the bacteria become resistant to three or more classes of antibiotics, they may be considered multidrug-resistant (MDR) bacteria. If the bacterial strains may develop resistance to one or two groups of antibiotics, then they may call extensively drug resistant (XDR—a bacterium resistant to most of the treatment drug options and susceptible to only a few options) (Moghadam et al., 2020), while if the bacterial strains develop resistant against all available antibiotics, then they are classified as pan-drug resistant (PDR—resistant to all drugs from all classes and susceptible to none of the antibiotics) bacteria (Abat et al., 2018; Weinberg et al., 2020). For example, MDR *Acinetobacter* species isolates are resistant to at least three antimicrobial classes (penicillins, cephalosporins, aminoglycosides, and quinolones) (Palavecino and Kilic, 2026; Clark et al., 2016). XDR *Acinetobacter* species isolates developed resistance against all classes described above, while the PDR *Acinetobacter* species includes XDR *Acinetobacter* isolates that are resistant to polymyxins and tigecyclines (Ali, 2020; Rossi et al., 2025). The microbial infections have increased drastically over the last few decades. Continuous deployment of antimicrobial drugs in curing various infectious diseases has led to the emergence of resistance among different strains of bacteria. Multidrug resistance (MDR) can be characterized as microbial resistance to certain administered drugs despite their earlier sensitivity (Ge M. et al., 2025; Tosas Auguet et al., 2025). These resistant microbes have the capability to combat the effect of antimicrobial drugs, leading to futile treatment, which may result in persistence and dispersal of infections. Although MDR development is a natural phenomenon, many with immunocompromised conditions, such as organ transplantation patients, diabetic patients, HIV-infected patients, and patients with severe burns, are more prone to hospital-acquired infectious diseases, which contribute to further spread of MDR. According to the World Health Organization (WHO), several bacteria show severe resistance against various antibiotics, such as *Escherichia coli* against cephalosporins and fluoroquinolones, *Staphylococcus* aureus against methicillin, *shigella* species against fluoroquinolones, *Klebsiella pneumoniae* against antibiotics carbapenems and cephalosporins, streptococcus pneumoniae against penicillins, and *Mycobacterium tuberculosis* against isoniazid, rifampicin, and fluoroquinolones causing blood, urine tract infections, and pneumonia (Romyasamit et al., 2024). MDR has become a serious dilemma for public health globally. The chances of controlling tuberculosis have been restricted due to the *Mycobacterium tuberculosis* bacterium's resistance against its respective antibiotics, thereby making it a serious concern worldwide. A survey conducted in 2012 reported MDR in 6% of newly diagnosed TB cases and 20% formerly treated TB cases. Moreover, around the globe, 92 countries were found to have extensive drug resistance against TB (Rhodes, 2025). MDR is classified as primary resistance that occurs when bacteria have never encountered the drug in a specific host, and secondary resistance arises in the bacteria after exposure to the drug (Xiong and Zhong, 2026; de Greeff et al., 2020).

Over recent decades, there has been a substantial increase in the global burden of multidrug-resistant (MDR) infection, posing a serious threat to public health worldwide. According to WHO, pathogens such as *E. coli, Klebsiella pneumoniae, Pseudomonas aeruginosa, Acinetobacter baumannii*, and *Staphylococcus aureus* exhibit high-level resistance toward antibiotics, which resulted in noteworthy treatment challenges, such as reduced treatment options, enhanced treatment failure risk, and reliance on less effective or more toxic antibiotics. Moreover, the clinical infection management causes prolonged hospitalization, increased healthcare costs, and elevated mortality rates. This highlights the urgent need for antibiotic stewardship, improved surveillance, and novel antimicrobial strategies (Mallik et al., 2025).

## 3 Genes and bacterial drug resistance

The phenomenon of drug resistance emerged even before the characterization of the first antibiotics, penicillin. The excessive use of antibiotics has further enhanced the emergence of resistance in many bacterial genera (Holmes et al., 2016; Naamala et al., 2016). The antibiotic resistance phenotypes appeared in microorganisms by mutation in chromosomal DNA through transformation, leading to the transfer and acquisition of new genetic material between the same or different species of bacteria, which produce mosaic proteins (Choe et al., 2025). Building upon the mechanistic framework of antibiotic resistance described in Section 2, the underlying genetic determinants provide the molecular basis for these resistance strategies. ARGs encode the function that enables bacteria to hinder antimicrobial activity, while their expression drives the physiological adaptations. Thus, resistance should be understood as a continuum connection between genetic determinants to functional cellular response (Coluzzi and Rocha, 2025).

### 3.1 Functional organization of resistance genes

Antibiotic-resistant genes can be grouped according to their encoding resistance mechanisms. These include (i) the gene responsible for enzymatic degradation or antibiotic modification (e.g., beta-lactamases, aminoglycoside-modifying enzymes), (ii) genes mediating alteration of antibiotic targets (e.g., gyrA, rpoB, and pbp gene mutations), and (iii) efflux system-encoded genes that actively remove antimicrobial agents (e.g., tetA, acrB, and mexAB). All these directly correspond to the major mechanism of resistance, reinforcing genotype to mechanistic linkage (Coluzzi and Rocha, 2025).

### 3.2 Regulation of resistance gene expression

The activity of ARGs is controlled via regulatory elements, such as operons, promotors, and global transcriptional regulators. Regulatory proteins modulate gene expression in response to environmental cues such as those from TetR, MarR, AraC, and SOxS families. This ensures that resistance genes are expressed only when required, reducing metabolic burden while increasing survival under antibiotic stress (Li et al., 2022).

### 3.3 Environmental induction and adaptive response

ARGs expression is majorly responsive to environmental conditions. Antibiotic exposure can induce expression of AR genes and activate stress response pathways, even at sub-inhibitory concentrations. Additional triggers, such as nutrient limitation, oxidative stress, and host-derived signals, further mediate genetic activity. These environmental inputs not only increase survival but also promote HGT, accelerating the resistance spread within and between bacterial populations (Coluzzi and Rocha, 2025; Li et al., 2022). Recent studies further highlight environmental reservoirs of resistance, such as microplastic-associated biofilms, which act as hotspots for antibiotic resistance gene (ARG) accumulation and horizontal transfer. These environments facilitate close microbial interactions, accelerating ARG dissemination across diverse bacterial communities and linking environmental pollution to clinical resistance emergence (Zhang et al., 2025).

### 3.4 From gene to physiological resistance phenotypes

The functional significance of ARGs lies in their ability to generate notable physiological outcomes. As discussed in Section 5, gene expression translates into adaptive cellular responses that determine resistance phenotypes collectively, for instance, β-lactamase genes, enzymatic degradation of β-lactam antibiotics, and sustained bacterial growth under antibiotic exposure; Efflux pump genes, decreased intracellular drug accumulation, multidrug resistance, and persistence; and target-modifying mutations (e.g., gyrA, rpoB), reduced antibiotic binding, and diminished therapeutic efficacy. These interactions depict that resistance is not solely defined by gene presence but also by their modulated response and physiological impact (Whittle et al., 2024). A comprehensive overview of major ARGs and their associated antibiotics has been tabulated here (Table 1).

**Table 1:** Antibiotic resistance genes along with their associated drugs.

Antibiotic	Antibiotic-resistant genes	References
Aminocoumarins	GyrB, ParE, ParY, alaS, cysB	Xie et al., 2025; Mancini et al., 2025; Weddeling et al., 2025; Shirakawa et al., 2020
Aminoglycosides	Genes coding for aminoglycoside acetyltransferases—ACC genes such as AAC(3), AAC(2′), AAC(1), AAC(6′), cpa and acetyltransferase, amp acetyltransferase, AAC(6′)-Ib-cr	Sanz-García et al., 2019; Thacharodi and Lamont, 2022; Pawlowski et al., 2016; Feßler et al., 2018; Bordeleau et al., 2021; Belotindos et al., 2021; Cox et al., 2015
	Aminoglycoside nucleotidyltransferases (ANT) genes: ANT(3″), ANT(2″), ANT(6), ANT(9) ANT(4′)	Mancini et al., 2025; Weddeling et al., 2025; Shirakawa et al., 2020; Sanz-García et al., 2019; Thacharodi and Lamont, 2022; Pawlowski et al., 2016; Feßler et al., 2018
	APH (Aminoglycoside phosphotransferases), APH(9)APH(6), APH(3″), APH (3′) APH(4), APH(2″), APH(7')	Bordeleau et al., 2021; Belotindos et al., 2021
	16S ribosomal RNA (rRNA) methyltransferases RmtA, RmtB, RmtC, ArmA, Sgm	Cox et al., 2015; Goudarzi et al., 2020; Hu et al., 2025
β-Lactams	Class A β-lactamases{IMI, CTX-M, GES, KPC, PER, VEB, SME, TEM, AER, CARB, SHV-LEN, BEL, OXY, OKP, ROB, CblA, CfxA, TLA, BlaA, cepA, Sed, NmcA, VCC, blaZ, R39, LRA, BlaA, blaF, EXO, RCP, ACI, HERA, RSA, ARL, AST, BCL, BES, BIC, BKC, BRO, CBP, CGA, CIA, CKO, CME, ERP, DES, FONA, FAR, FPH, FRI, LAP, FTU, SCO, GPC, blaS, AAK, AXC, CRH, CRP, CSP, GIL, KLUC, LRG, LUS, LUT, MAL, OHIO, ORN, PAU, PLA, PME, PSV, RAHN, RSA2, RUB, SFC, SFO, SGM, SPU, TER, VHH, VHW}	Cox et al., 2018; Bassenden et al., 2016; Chen et al., 2013; Martí et al., 2019
	Metallo-β-lactamases, class B, {BlaB, VIM, IMP, NDM, CcrA}	Rahman et al., 2015; Doi et al., 2016
	β-Lactamases, class C, {ACT, CMY, AmpC, LAT, PDC, ACC, BIL, BUT, CFE, CMG, DHA, FOX, LEN, MIR, MOR, OCH, OKP-A, OKP-B, OXY, TRU, ZEG, cepH}	Wachino and Arakawa, 2012; Bush, 2018
	Class D β-lactamases {OXA, LCR, NPS, LRA, BAT, BPU, BSU, CDD, RSD2}	Usman Qamar et al., 2020; Pillai et al., 2011; Mercuri et al., 2020; Berglund et al., 2021; Grigorenko et al., 2022; Ibrahim, 2022; Philippon et al., 2022
Chloramphenicol	Chloramphenicol acetyltransferase [cat A1, catA2, catA3, catA4, catA5, cat86, cat (pC221), cat(pC223), cat(pC194), catP, catS, catA13, catB, catA14, catQ, catB1, catB2, catB3, cmlA, floR, fexA, cmr, cfr, pexA]	Evans and Amyes, 2014; Tawfeeq et al., 2020; Ren et al., 2023; Gudeta et al., 2016; Dahar et al., 2024
	Chloramphenicol phosphotransferase	
Glycopeptides	Van-A, Van-B, Van-C, Van-D, Van-E, Van-F, Van-G, Van-I, Van-M, Van-N, Van-L, Van-O, Van-R, Van-S	Tirumalai and Fox, 2013
Macrolides	MefE, MefA, Mel, ErmA, ErmB, Erm (31), Cfr 23S rRNA methyltransferase	Yoon and Jeong, 2020; Roberts and Schwarz, 2017; Parkhill et al., 2001; Bale et al., 2023
	Macrolide esterases EreA, EreB	
	Erm 23S rRNA methyltransferases	
	GimA, Mgt, Ole	
	Macrolide glycosyltransferases	
	Macrolide resistance efflux pumps	
	Macrolide phosphotransferases (MPH)	
Tetracyclines	Tetracycline inactivation enzyme TetX. TetM, TetO, TetQ, Tet32, Tet36. Tetracycline resistance major facilitator superfamily (MFS) efflux pumps. Mutant porin PIB (*por*) with reduced permeability. TetA, TetB, TetC, Tet30, Tet31, Tetracycline resistance ribosomal protection proteins	Stogios et al., 2016; Akrami et al., 2019; Selim, 2022; Golkar et al., 2018
Rifampin	Rifampin ADP-ribosyltransferase (Arr), Rifampin phosphotransferase, Rifampin glycosyltransferase	Morar et al., 2012; Li et al., 2015
	Rifampin monooxygenase, Rifampin-resistant beta-subunit of RNA polymerase (RpoB). Rifampin resistance RNA polymerase-binding proteins, DnaA, RbpA.	
Quinolones	Fluoroquinolone-resistant GyrA, GyrB, ParC, fluoroquinolone acetyltransferase, fluoroquinolone-resistant DNA topoisomerases, quinolone resistance protein (Qnr) *qnrA, qnrB, qnrC, qnrD*, and *qnrS*	Koraimann, 2018; Sheykhsaran et al., 2019; Elshamy and Aboshanab, 2020; LaPlante KL, 2022; Warburton et al., 2016; Surette et al., 2021
Streptothricin	Streptothricin acetyltransferase (sat)	Van Hoek et al., 2011; Jacoby et al., 2015
Sulfonamides	Sulfonamide-resistant dihydropteroate synthases, Sul1, Sul2, Sul3, sulfonamide-resistant FolP	Yanat et al., 2017

## 4 Genetic elements and plasmids associated with drug resistance

The bacterial resistant strains are an increasing threat to human health. Resistance mechanisms to evade the toxic antimicrobial action have been recognized for all known antimicrobials that are currently available for clinical use. The mutation in normal cellular genes or the acquisition of foreign resistance genes or the combination of both mechanisms results in acquired antibacterial resistance (Belay et al., 2024). The bacteria can resist a particular antibiotic via degradation of enzymes or change of antimicrobial, target site mutation, or alter bacterial cell wall permeability toward antibiotics, or active efflux of antibiotics across the cell membrane (Nikolic and Mudgil, 2023). The mobile genetic elements, such as transposons, plasmids, and integrons, contribute to the rapid antibiotic resistance spread among various bacterial genera. The antimicrobial resistance genes that gather on these mobile elements lead to conditions where MDR phenotypes may transfer to susceptible recipients through a single gene event (Tomova et al., 2018; Geetha et al., 2020). Acquired antibiotic resistance can be found in any region of DNA capable of transferring information from one part of the genome to another. Mobile genetic elements contain all the required genetic information from one bacterium to another, and conjugative elements, which transmit to another host using the combined actions of co-resistant conjugative elements, are the main players in HGT (horizontal gene transfer) (Redgrave et al., 2014; Dalhoff, 2012). Bacteriophages aid in the transmission of DNA across bacteria through the process of transduction, which involves the transfer of bacterial DNA contained in the phage head into the recipient bacterium. Integrons and transposons are elements capable of translocation to new places in the genome but not of transfer to new hosts (Burckhardt and Escalante-Semerena, 2019). Antibiotic resistance can be acquired by both Gram-positive and Gram-negative bacteria through metamorphosis into competent bacteria with the ability to accept DNA from the environment. Many streptococci, for example, are competent at a specific stage of their growth, whereas others have no discernible competence window. Some bacteria, such as Neisseria, require a specific sequence in order to successfully take up DNA, whereas others do not (Farley and Metcalf, 2019). During the transformation process, DNA picked up by recipient bacteria may be incorporated into the host genome via homologous recombination or transposition, whereas certain DNA, such as plasmids, can replicate autonomously (Sartori et al., 2026). Numerous studies have explored plasmid bacterium co-evolution (Maka et al., 2015), as well as the association between conjugative plasmids and antibiotic resistance genes spread in various bacterial families (Redgrave et al., 2014).

Plasmids are circular non-chromosomal components found in all bacteria with their own replication origin. HGT of plasmid-borne genes is responsible for the inclusive development of resistance (MacLean and San Millan, 2019). In addition, plasmids can also be transferred to new hosts through the conjugation process that dock conjugation genes, whereas mobilizable plasmids are those that hold the origin of transfer and use conjugative plasmids to relocate to new hosts. About 25% of plasmids are conjugative and self-transmissible, while 25% are mobilizable and encode a part of the conjugative machinery required to transfer other conjugative elements. However, the rest 50% of plasmids do not encode conjugative genes and are non-transmissible (Partridge et al., 2018; Botelho et al., 2019). The plasmids having a broad host range could be transferred to different species, while the narrow host range plasmids are restricted to transfer to one genus or species. However, plasmids that are unable to be moved through this mechanism are shifted via conjugative elements, resulting in transient fusions known as co-integrations. Plasmids promote the formation of cell contact by producing microfibrillar external covering substance influenced by pheromones. The mobilizable plasmids contain DNA transfer genes, essential for the structure and function of the relaxosome but not for mating pore formation. Some resistance plasmids are incompatible with each other in microbial cells, resulting in the formation of incompatibility groups (Sheppard et al., 2016).

The transposons, known as jumping genes, are defined as DNA sequences of mobile gene elements and were discovered in 1940 by Barbara McClintock. Few transposons are confined to the insertion site in a genome and are divided into two main classes: (1) retrotransposons, found in eukaryotes, (2) DNA transposon, found in prokaryotes as well as in eukaryotes (Dubnau and Blokesch, 2019). The bacterial transposons are the DNA transposons and the Tn family that are commonly additional gene carriers for antibiotic resistance.

Transposons can be transferred through plasmids to other plasmids, from a DNA chromosome to a plasmid, or from a plasmid to a DNA chromosome, which may be one reason for the transfer of antibiotic resistance gene (ARG) in bacteria (Bottery et al., 2019; Carattoli, 2013). Transposons are associated with an enzyme transposase (Tase), which is responsible for transposition (Shintani et al., 2015). In bacteria, the transposons are categorized into four groups known as insertion sequence, composite transposons, non-composite transposons, and transposable phage Mu (Garcillán-Barcia and Rocha, 2025). The insertion sequence can cause bacterial drug resistance in a variety of ways (Ramsay et al., 2016; Guilcazo et al., 2025). Insertion sequences can inactivate genes in the insertion site through direct integration, while composite transposons can transmit antibiotic resistance genes to other bacteria. Aminoglycoside resistance is caused by IS256, which is found in Tn4001's composite transposon. By harboring additional resistance genes, composite and non-composite transposons can improve bacterial drug resistance (Babakhani and Oloomi, 2018; Stuart et al., 2025; Gozukirmizi et al., 2016). The Gram-negative bacterial antibiotic resistance is formed by non-composite transposons (Makałowski et al., 2012). Bacterial species can transmit transposons via plasmids, bacteriophage, and it has been reported that the drug resistance genes are transmitted through DNA phage by HGT (Katahira et al., 2024; Fowler and Hanson, 2014). Tn (5,9,10) are some of the composite transposons found in Gram-negative bacteria, while Gram-positive bacteria contain Tn4001 and Tn4003 transposons. Gram-positive bacteria Tn551, Tn917, and Tn4451 complex resistant transposons, as opposed to Gram-negative bacteria's Tn (1,3,21,501,721,3926). These components have the ability to jump within the DNA molecule or from one DNA molecule to another (Ross et al., 2021).

Integrons are ancient structures that contribute to bacterial evolution by acquiring, storing, disposing, and restoring reading frameworks in cassettes, which are mobile elements found on around 17% of bacterial chromosomes (Ghaly and Gillings, 2022). These elements have a vital function in the resilience and fitness quality of bacteria, which allows them to survive in a variety of settings. Integrons are referred to as a group of genetic elements capable of capturing gene cassettes, though in the bacterial genome, around one-third of integrons are discovered without gene cassettes (Colomer-Lluch et al., 2011). They have a significant role in the spread of antibiotic resistance, especially among Gram-negative bacteria. Integrons are the primary cause of MDR in Gram-negative bacteria (Pillai et al., 2011). Antimicrobial resistance is conferred by having resistance determinants such as ARG, mobilizing them as part of chromosomes and plasmids, and integrating them away from their source. They are classified according to the integrase amino acid sequence (Int1). Integrase class 1 (Int1), class 2 (Int2), and class 3 (Int3) were linked to mobile genetic elements, while Integrase 4 (Int4) is associated with chromosomal integrons (Dimitriu et al., 2021). Table 2 shows the frequent gene cassettes in different types of Integrons found in bacterial species.

**Table 2:** The common gene cassette arrays in types of integrons in various bacterial species and their role in antibiotic resistance.

Bacteria	Gene cassettes	Integrons class	Antibiotics associated with gene cassettes	References
*Escherichia coli*	dfrA1, dfrA5, dfrA7, dfrB2, dfrA12, dfrA17, dfrA14, aadA1, aadA2, aadA5, aadB, dfrA1-aadA1, dfrA1-gcuC, dfr17-aadA5, dfrA1-sat2-aadA1, dfr12-gcuF-, dfrA1-sat1-aadA1, estX-sat2-aadA1, ^bla^OXA-101-aac(6′)-Ib, ere2.	Int1, Int2, Int3	Extended Spectrum beta-lactamase, aminoglycosides, erythromycin, trimethoprim.	Osagie and Olalekan, 2019; Sütterlin et al., 2020; Sultan et al., 2018; Moyaert et al., 2017; Márquez et al., 2008
*Salmonella* species	dfrA1, dfrA7, aadA, aadA1a, aadA2, aadA5, aadB, dfrA17, dfr12-gcuF-aadA2, dfrA1-aadA1a, dfrA1-gcuF, dfr17-aadA5, bla_CARB − 2_	Int1, Int2	Extended spectrum β-lactamases, aminoglycosides, and trimethoprim.	Kargar et al., 2014; Zhang et al., 2019; González-Villalobos et al., 2022
*Acinebacter baumannii*	bla_CARB − 2_, sat1, aadB, aadA1, dfrA1, aadA2, dfrA7, dfrA1-gcuF, dfr17-aadA5, dfrA1- aadA1, dfr12-gcuF-aadA2.	Int1, Int2	Extended spectrum beta-lactamase, aminoglycosides, trimethoprim.	Asgharpour et al., 2014; Torres-Elizalde et al., 2021; Monte et al., 2020; Domingues et al., 2015
*Klebsiella* species	bla_CARB − 2_ bla_GES − 1_, aad (A, A1), aadB, dfrA1-aadA1a, dfrA7, dfrA1, dfr17-aadA5, dfr12-gcuF-aadA2, dfrA1-gcuF.	Int1, Int2, Int3	Extended spectrum beta-lactamase, trimethoprim, aminoglycosides	Liu et al., 2014; Akrami et al., 2017; Nikibakhsh et al., 2021
*Staphylococcus aureus*	aad (A1,A2), dfr12-gcuF-aadA2, aacA4-cmlA1, dfr17-aadA5	Int1	Chloramphenicol, trimethoprim, aminoglycoside.	Malekjamshidi et al., 2020; Manohar et al., 2018
*Pseudomonas aeruginosa*	dfr12-gcuF-aadA2, aadB, aadA2, dfr17-aadA5.	Int1	Trimethoprim, aminoglycosides.	Wu et al., 2013; Mahmoud et al., 2021
*Enterobacter* species	AadA2, dfrA7, aadA1a, dfr17-aadA5, dfrA1-aadA1a, dfr12-gcuF-aadA.	Int1	Aminoglycosides, trimethoprim	Goudarzi et al., 2017; Ghaly et al., 2020
*Enterococcus faecalis*	Dfr12-gcuF-aadA2, aadA1a, dfrA1-sat1-aadA1	Int1	Aminoglycosides, trimethoprim	Ahmadian et al., 2020

## 5 Physiological adaptations in bacteria leading to drug resistance

The ability of mobile genetic elements containing antibiotic resistance genes to spread is modulated by a range of factors, such as selective pressure in the environment, host factors, and properties of genetic elements themselves. Numerous protective systems exist in bacteria that protect them from incoming DNA, such as modification systems and CRISPR-Cas systems (Kaushik et al., 2018). These systems are mechanistically complex at the same end point of recognizing and evading foreign DNA. They work via identifying specific sequences in incoming DNA that are not protected by methylation and digestion (Mortazavi et al., 2018). Importantly, resistance phenotypes emerge from dynamic interactions between genetic determinants and environmental stimuli, rather than isolated gene effects. Gene regulatory networks modulate the expression of resistance-associated pathways such as efflux, stress response, and metabolic adaptation in response to exposure to antibiotics. This systems-level integration highlights that physiological resistance should be interpreted as a context-dependent expression of genomic potential, shaped via intrinsic regulatory circuits and external selective pressure (Thu et al., 2019). Beyond planktonic resistance mechanisms, bacteria frequently adopt biofilm-associated lifestyles, which integrate genetic regulation and physiological adaptation to enhance survival under antimicrobial pressure.

Multiple pathways are involved in causing resistance against a particular antimicrobial group; for example, the resistance to fluoroquinolones is achieved via three mechanisms. First, gene mutation producing a target site; second, efflux pump over-expression that expels the drug from the cell; and third, target site protein protection. However, bacterial species seem to acquire a preference for one mechanism over the other. In Gram-negative bacteria, the β-lactamase is the common route for β-lactam resistance, whereas Gram-positive bacterial resistance is developed by modifying the target molecules, penicillin-binding proteins (PBPs). These mechanisms are thought to be due to a significant difference in the cell envelope between Gram-negative and Gram-positive bacteria. The presence of an outer membrane in Gram-negative bacteria allows antibiotic penetration into the periplasmic region to be controlled. Certain porins are required by the β-lactams to reach PBPs in the inner membrane, limiting the access of drug molecules to the periplasmic region and allowing β-lactamase synthesis at adequate concentrations (Munita and Arias, 2016).

### 5.1 Modification or inactivation of antibiotic molecules

**Figure 2:**
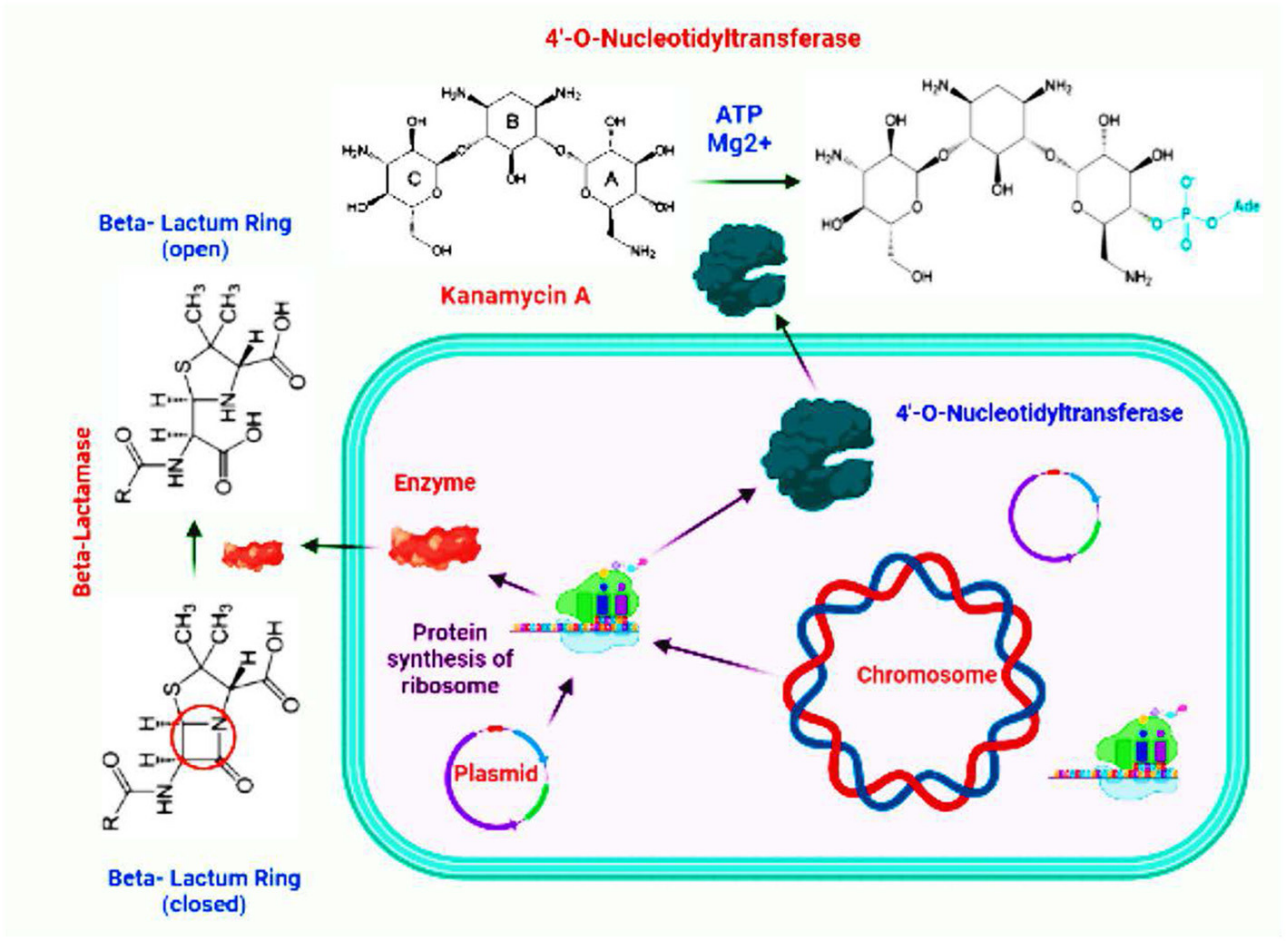
Modification of an aminoglycoside (Kanamycin A) by 4′-*O*-nucleotidyltransferase and opening of the β-lactam ring of an antibiotic by the activity of β-lactamase.

Bacteria can produce inhibitory enzymes, which may add chemical moieties to antibiotics, or they can degrade the drug molecule, preventing the antibiotic from interacting with its target. The most well-known acquired antibiotic resistance mechanism in both Gram-positive and Gram-negative bacteria is the production of enzymes capable of introducing chemical modifications to antimicrobial molecules. The majority of antibiotics that affect these enzymatic changes function by inhibiting the synthesis of proteins (Wilson, 2014). Many modifying enzymes are involved in catalyzing the reaction of acetylation, phosphorylation, and adenylation. Besides biochemical reaction, the steric inhibition is also related to the ultimate effect that reduces the avidity of the drug for its target. The second mechanism of resistance involves the destruction of the antibiotic molecule. For example, beta-lactam resistance via beta-lactamase relies on the destruction of the antibiotic compound. The breakdown of the enzyme β-lactamase occurs at the amide bond of the β-lactam ring. After penicillin became widely available, *Staphylococcus aureus* infection became clinically relevant, and the resistance mechanism was discovered to be plasmid-encoded penicillinase, which was easily transmitted between *Staphylococcus aureus* strains, resulting in rapid dissemination of the resistance trait (Naamala et al., 2016; Bush, 2013; D'Costa et al., 2011). Degradation or modification of antibiotics such as β-lactams and aminoglycosides by bacterial lactamases and hydrolases has been a well-established strategy in bacterial resistance. β-Lactamases are the enzymes that open the core functional β-lactam ring of cephalosporins and penicillins. In recent years, a new enzyme variant NDM (New Delhi metallo-β-lactamase) has been identified that breaks the β-lactam ring of carbapenems. More than 20 variants of NDM have been identified, which have shown resistance against a spectrum of antibiotics (Dortet et al., 2014; Nordmann et al., 2011). In addition to lactamases, there are several bacterial enzymes that can introduce different groups to the structure of antibiotics and modify them to make them unable to bind the target molecules. Amide, phosphate, acetyl and hydroxyl, and sugar groups are often introduced to many antibiotic molecules for their modification (Naamala et al., 2016; Liu et al., 2017; Yao et al., 2017). Modification and inactivation of two antibiotics by the action of typical bacterial enzymes have been illustrated (Figure 2).

### 5.2 Reduction in permeability

**Figure 3:**
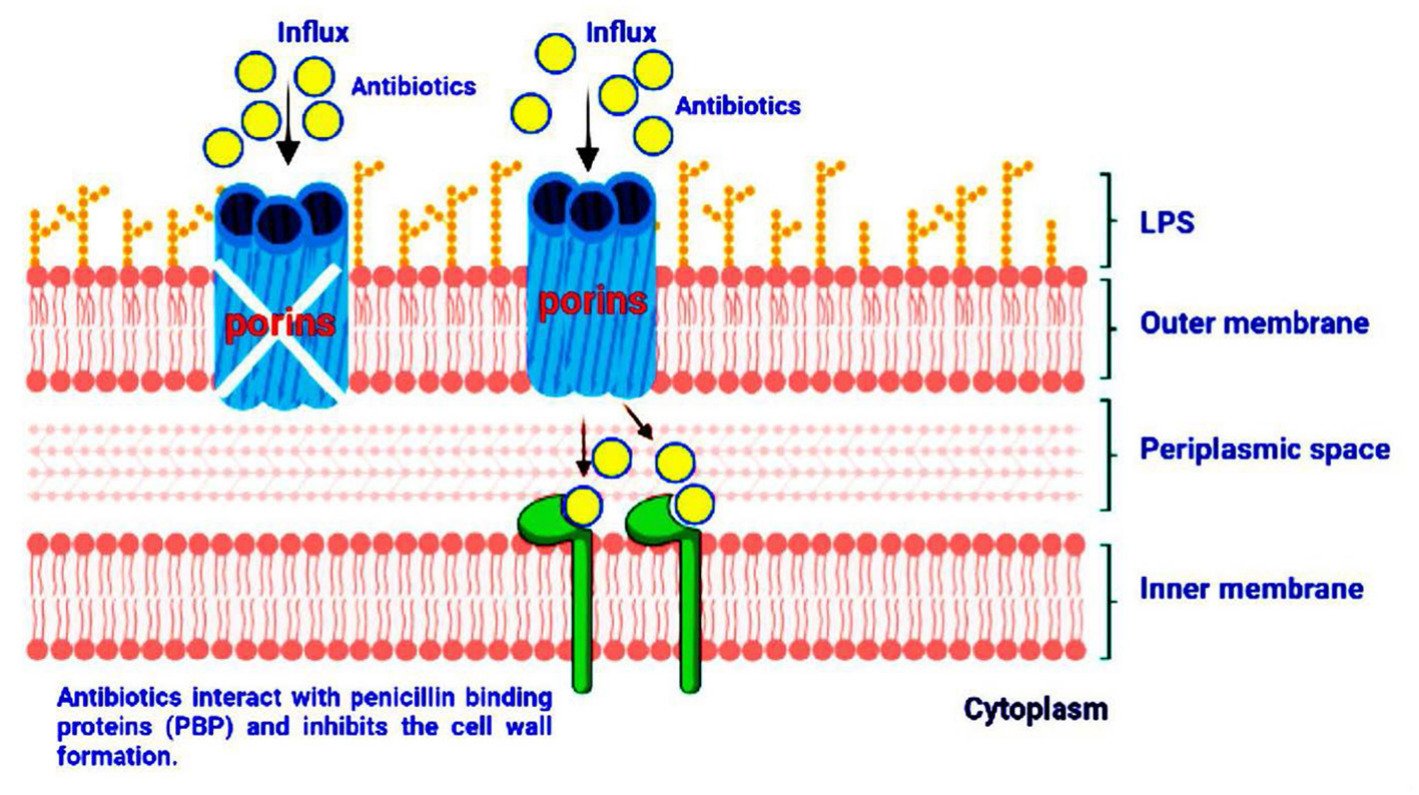
Influx of antibiotic molecules through porins by simple diffusion. Reduced expression or loss of active conformation of porins leads to the inhibition of antibiotic intake.

In the cytoplasmic membrane, several drugs have intracellular bacterial targets. The drug must enter the outer or/and the cytoplasmic membrane to exert its antibacterial effect. Bacteria have evolved by reducing the absorption of antimicrobial molecules to prevent the antibiotic molecules from reaching their periplasmic or intracellular targets. This process is important in Gram-negative bacteria as it restricts the drug inflow. The outer membrane acts as the first line of defense for many hazardous substances, such as antibacterial agents. The hydrophilic compounds frequently use water-filled diffusion channels known as porins for their transverse, are particularly affected by changes in membrane permeability, for example, fluoroquinolones, beta-lactams, and tetracyclines (Yoon et al., 2017). The ineffectiveness of vancomycin against Gram-negative bacteria due to its inability to penetrate the outer membrane is a prime example of this natural barrier's efficacy. Similarly, *Pseudomonas* and *Acinetobacter baumanii*'s resistance to beta-lactam (as compared to enterobacteriaceae) can be explained by a reduced number or varied expression of porins (Winterhalter, 2021). Porins have been categorized into several categories based on their structure (trimeric vs. monomeric), selectivity, and expression regulation. The three primary proteins produced by *E. coli* (known as OmpF, OmpC, and PhoE) and the *P. aeruginosa* OprD are classic instances of porin-mediated antibiotic resistance among the best-characterized porins. Porin changes can be caused by three different mechanisms: first, by the change in type of expressed porins; second, by the level of porins expression; and third, porin function degradation. Prominently, permeability changes occur by any of these pathways, which typically lead to low-level resistance and are frequently linked to other resistance mechanisms, that is, increased expression of efflux pump (Ude et al., 2021) (Figure 3).

### 5.3 Efflux pumps in drug resistance

Bacterial machinery that secretes harmful chemicals from the cell can lead to the development of resistance to antimicrobials. In the early 1980s, the identification of the first efflux mechanism capable of pumping tetracycline out of cytoplasm of *E. coli* was reported. Numerous efflux pumps have been discovered since then in both Gram-negative and Gram-positive bacteria. These systems may be substrate specific or a broad spectrum of antimicrobial classes (Xu et al., 2026). Efflux pump genes can be found on the chromosome or in MGEs (mobile genetic elements). Furthermore, chromosomally encoded pumps may explain the reason for built-in resistance to specific drugs, for example, *E. faecalis*' intrinsic resistance to streptogramin A (Poole, 2005; Fahim et al., 2025).

SMR is a class of small proteins with a conserved sequence of approximately 150 amino acids and four transmembrane helix structures (Yu et al., 2025). Mmr is the first SMR family efflux pump identified, displaying homology with QacEu and EmrE efflux pumps found in *Staphylococcus* and *E. coli*, respectively (Fernández and Hancock, 2012). These efflux pumps are classified as secondary active transporters. In 1980, Henderson and Maiden found MFS superfamily in xylose, galactose, arabinose, and lactose as the H^+^ symporter that resembles tetracycline transporters (TetA) that make bacteria resistant to tetracycline (Li and Nikaido, 2009). These transporters have structural and amino acid similarities between MFS and other transporters, and structurally different substrates originating from unrelated living organisms with similar homology are believed to be MFS members (Chen et al., 2015; Al-Majdoub et al., 2025; Varela et al., 2023). Plant, fungal, bacterial, and human transporters are all included in this family, which has a common transport mechanism (Krammer and Prévost, 2019; Arafa et al., 2021). MFS is a group of 74 protein families, the majority of which are composed of single polypeptide chains having transmembrane regions. Almost half of efflux proteins are members of the MFS superfamily (Ge Y. et al., 2025). MFS encodes for both inherent and acquired antibiotic resistance in both types of bacteria (Nikaido and Pagès, 2012). This superfamily member obtains energy via proton gradient (Kumar et al., 2013). Both susceptible and resistant bacteria include efflux genes encoding transporters (Ge Y. et al., 2025). These efflux pumps are classified as secondary active transporters. MFS transporters are cataloged in a database and further classified according to their sequence structure and homologous transporters (Saier et al., 2014). Multidrug and toxic compound extrusion (MATE) family of transporters is a member of a new superfamily and can be found in humans, plants, and animals. NorM from *Vibrio parahaemolyticus* was the first MATE transporter discovered (Majidinia et al., 2020). The energy source for these transporters is the gradient of H, Na, or both. Efflux pump genes of MATE have not been studied in their native organisms but have been cloned from many pathogens. These efflux groups are secondary active transporters. ATP-binding cassette (ABC) superfamily derives its energy from ATP hydrolysis. The most important efflux pump is P-glycoprotein, which consists of two nucleotide-binding domains (NBD-1 and NBD-2) and two hydrophobic transmembrane domains (TMD-1 and TMD-2) (Elbahnsi et al., 2026; Yan, 2015). These efflux pumps constitute the primary active transporters. Resistance-nodulation-division (RND) family members play an important role in Gram-negative bacteria, which provides intrinsic antibiotic resistance. They have tripartite organizations that result in the expulsion of a broad spectrum of antibiotics. In *E. coli*, the chief RND efflux pump is the AcrAB-TolC pump (Carbó and Rodríguez, 2023). In the RND family, AcrA, AcrB, and TolC are proton-dependent efflux pumps. AcrB is an inner membrane protein that is a homotrimer with a cap-like structure in each subunit (Hassan et al., 2015). In *Mycobacterium* TB, the representative RND family efflux pumps are mycobacterial membrane protein (MmpL) and mycobacterial small membrane protein (MmpS). Proteobacterial Antimicrobial Compound Efflux (PACE) of efflux pumps are secondary active transporters. This is a newly discovered efflux pump group found in some Gram-negative bacteria. Very little information is known about PACE and its resistance mechanisms, and it is known that they confer resistance to various biocides (Sun et al., 2014). PACE family members' efflux pump genes have highly conserved amino acids in the core of the bacterial genome.

**Figure 4:**
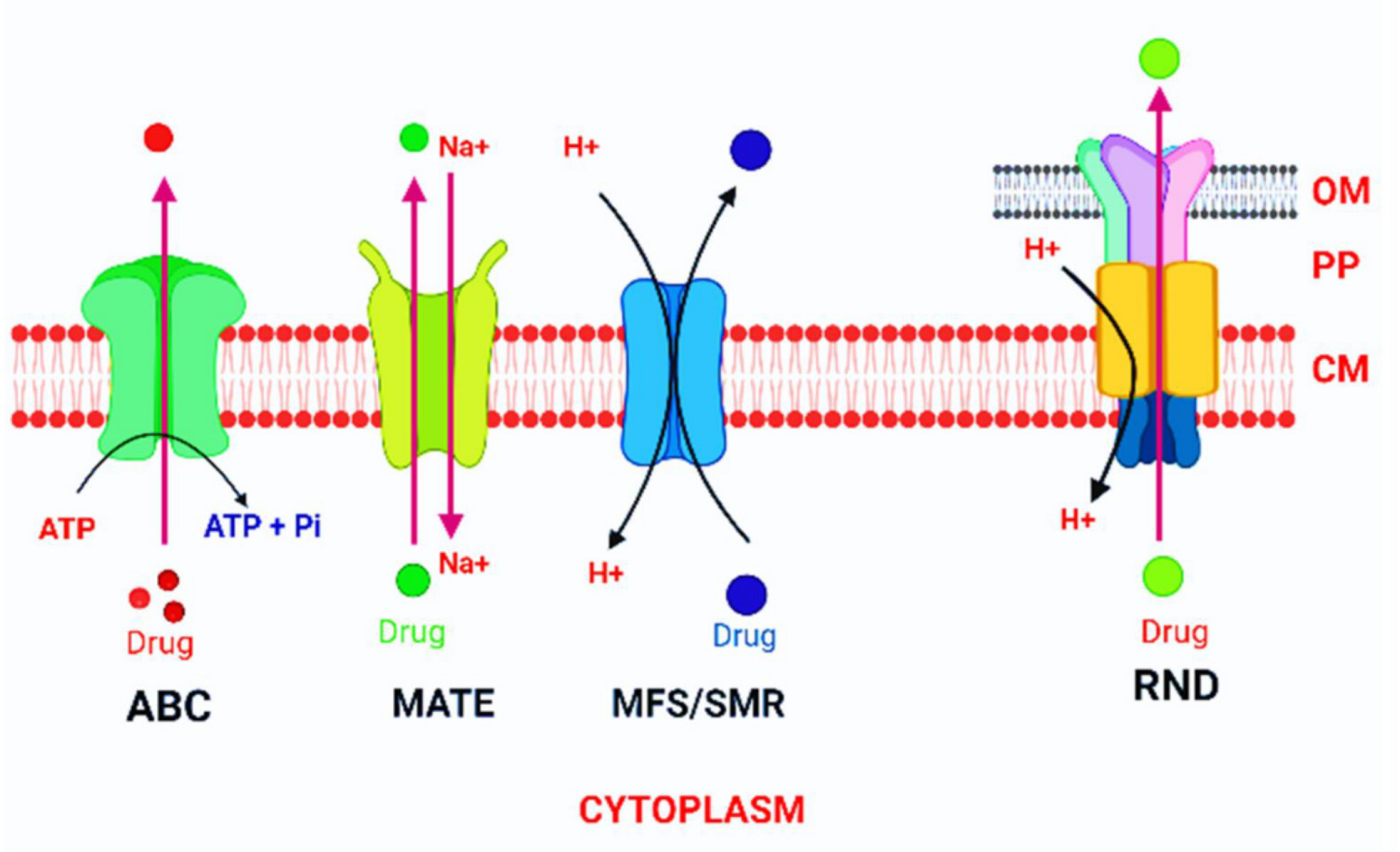
SMR MATE, MFS, ABC, and RND are the six families of transporter proteins identified in bacteria.

Overexpression of efflux pumps is a common cause of antimicrobial treatment failure (Ebbensgaard et al., 2020; Hastert et al., 2025). Tetracycline resistance was the first EP-based bacterial resistance discovered in *Escherichia coli* (Karam et al., 2016; French, 2010). EP overexpression can affect genes encoding antibiotic target sites (Adrizain et al., 2018). The efflux pump has been examined in MDR Gram-positive and MDR Gram-negative species. The lack of novel antimicrobial medications under development is becoming an issue (Butler et al., 2013; Wong et al., 2014). The interest in efflux pump inhibitors and efflux pump systems has been growing (Gill et al., 2015; Wright, 2016; Başaran and Öksüz, 2023). EPI is used instead of inhibitors or modulators because different modulators or inhibitors listed in the literature do not act on the pump itself. Most efflux pump genes, on the other hand, are chromosomally distributed, and 3–12% of open reading frames are expected to encode membrane transport proteins (Ren and Paulsen, 2005; Hernando-Amado et al., 2016). These genes have highly conserved structures and are tightly regulated. Though these are expressed at the basal level, they are normally in a repressed form, lacking an effector or promoter activation (Rodrigues et al., 2012). Over-expression of efflux pumps is usually conferred clinically substantial drug resistance, which may be temporary due to sub-inhibitory exposure to an antibiotic (Maor et al., 2025). The four major regulatory protein families involved in MDR efflux pump transcriptional control are AraC, MarR, MerR, and TetR (Huang et al., 2025; Alvarez-Ortega et al., 2013). MDR bacteria have been detected in bacteria isolated from an environment lacking new antibiotics (Alhusseini et al., 2026). Despite being at under less antimicrobial pressure than in a clinical setting, numerous soil-associated microorganisms and plants have a great number of efflux proteins (Blanco et al., 2016; Dallaev et al., 2025). This notion is supported by the discovery of *P. aeruginosa* strains with pumps capable of extruding fluoroquinolone drugs before their discovery (Ruggerone et al., 2013). Some efflux proteins confer specific antibiotic resistance, e.g., MexCD-OprJ in *P. aeruginosa* or TetM in *E. coli*, whereas some others expel a broad spectrum of antimicrobials, for example, NorA in *Staphylococcus aureus*, AdeABC in *A. baumanii*, or AcrAB-TolC in *E. coli*. Numerous investigations have shown the transporters' substrate specificity promiscuity (Yu et al., 2025). Biocides present in the pump substrate profile may induce overexpression of these pumps, resulting in resistance to all other pump substrate, including antibiotics (Buffet-Bataillon et al., 2016; Amaral et al., 2014). All bacteria have multi-drug transporters, which play a role in antibiotic resistance as well as their normal functions (Kaushik et al., 2018). Bacterial efflux pumps transfer substances over a bacterium's plasma membrane (Sinha et al., 2025). SMR MATE, MFS, ABC, and RND are the six families of transporter proteins identified in bacteria (Figure 4) (Birlutiu and Birlutiu, 2025; Opperman and Nguyen, 2015).

The proteobacterial antimicrobial compound efflux (PACE) was recently identified as a sixth bacterial efflux pump group (Poole, 2005). Efflux pumps are important in bacteria because they remove and eliminate toxic chemicals from bacteria before they reach their intracellular targets, thus, they serve as a protective function in bacteria (Karam et al., 2016). In order to combat antibiotic resistance, it is necessary to decrease efflux pump activity, which removes harmful chemicals from bacterial cells and is a key element in antibiotic resistance (Bohnert and Kern, 2016; Nasershariati et al., 2025). Drugs can be restored to their sensitivity by raising the intracellular concentration and decreasing intrinsic resistance with efflux pump inhibitors (EPIs). A key purpose for the development of EPIs is to produce small molecules that have the ability to attach conventional antibiotics to avoid MDR efflux systems. While in 2001, PAN was discovered as an EPI compound capable of inhibiting the therapeutically important *P. aeruginosa* efflux pumps, it has now been discovered to be an inhibitor of RND efflux pumps of other Gram-negative bacteria (Elbaiomy et al., 2025). MC-207 and MC-110 compete for extrusion with EB rather than blocking pumps. Although PAN is not an inhibitor of an efflux pump, it competes with other efflux pump substrates for expulsion, as demonstrated by Martins and colleagues using the Michaelis–Menten formula (Gao et al., 2025; Kinana et al., 2016; Mahamoud et al., 2007). Kinetic characteristics and their link to efflux pump structure are critical to designing successful EPIs (Gu et al., 2025). To find new EPIs, one of the most important concerns is to understand how EPIs prevent the transport of medications out of cells. Bacterial inhibitors may target genes that promote MDR, lowering the energy required by efflux pumps, preventing their functional assembly, or completely blocking the outer membrane channel (Yewale et al., 2020). To better understand the ligand-binding process in efflux pumps (such as AcrB), molecular simulations may be useful. When used on their own, EPIs should be ineffective against bacteria, but when combined with antibiotics, they should be more effective. Chemically, EPIs include molecules derived from natural (plant) sources, derivatives of current efflux inhibitors that are semi-synthetic, and new EPIs that are completely synthetic (Gordya et al., 2017).

### 5.4 Biofilm-mediated antibiotic resistance

Biofilm-associated infections are clinically significant, indicating that upto 60%−90% of chronic infections involve biofilm-forming bacteria, specially among ESKAPE pathogens (Ugwu et al., 2025). Biofilms are illustrated to be a major contributor to antibiotic resistance, characterized by structured communities of microbes embedded within a self-produced extracellular polymeric substance (EPS) matrix. The development of biofilm occurs via a sequential process involving initial attachment, formation of a micro-colony, maturation, and eventually dispersion, enabling bacteria to establish persistent infections in diverse environments (Flemming et al., 2023; Iaconis et al., 2024). Biofilm-associated resistance is modulated through several mechanisms. The EPS matrix acts as a physical barrier that limits antibiotic penetration, while metabolic heterogeneity within the biofilm results in slow-growing or dormant cells that are less susceptible to antimicrobial agents. Additionally, persister cells contribute to chronic and recurrent infections, and efflux pump expression is often upregulated in the biofilm state. Increased HGT within biofilms then further promotes the ARGs dissemination across the bacterial population (Lu et al., 2025; Qureshi et al., 2026). The formation and maintenance of biofilms are closely regulated through genetic networks, including quorum-sensing systems, such as luxS, las, and rhl, as well as biofilm-associated genes, such as bap, psl, and pel. These regulatory pathways coordinate bacterial behaviors and facilitate adaptation under environmental stress conditions (Singh et al., 2025; Dutt et al., 2022). Biofilm-associated resistance presents significant therapeutic challenges, as bacteria within biofilms can exhibit up to 1,000-fold enhanced tolerance to antibiotics as compared to planktonic cells, which leads to difficulties in eradication, increased risk to treatment failure, and the need for alternative strategies, such as anti-biofilm agents, novel drug delivery approaches, and combination therapies (Singh et al., 2025; Dutt et al., 2022).

## 6 Combating strategies against bacterial antibiotic resistance

Antibiotic resistance of microorganisms is becoming a global problem and has emerged because of increased antibiotic use, jeopardizing the therapeutic effectiveness of medications. Techniques that materialize ideally among the various ways utilized include designing antimicrobial peptides with multiple targets (Hassan et al., 2012), nanoparticles (NPs) for long-term and controlled drug release, AMPs and essential oils, phage therapy, combination therapy, liposomes as drug targeting vehicles, natural substances such as flavonoids, alkaloids, and antimicrobial modification. Strategies such as the development of molecules that hinder bacterial attachment to surfaces and target bacterial virulence factors, as well as contributing to protection through the production of inactivating antibodies, appear to be viable options for dealing with the threat of drug resistance (Kościuczuk et al., 2012). The strategies to combat the menace of antibiotic resistance are shown in Table 3.

**Table 3:** The combating strategies for antibiotic resistance.

Strategy	Entity	Explanation	Effect	References
Antimicrobial peptides	Bacteriocins	Peptides with 20–50 amino acids, both of which are cationic and amphiphilic. Their interactions with the negatively charged bacterial membrane result in the creation of transmembrane holes, which allow cellular solutes to leak out and finally kill the cell. Bacteriocins production genetic determinants are found on mobile genetic elements. The majority of Bacteriocins are found in *E. coli* and other enterobacteria.	Through cell wall biosynthesis inhibition, they target diseases such as *Clostridium* difficile and developing antibiotic-resistant bacteria, including MRSA, VRE, and enterohemorrhagic *E. coli, Staphylococcus aureus*, and *Staphylococcus epidermidis* are both susceptible to lysostaphin and bacteriocin.	Kaur et al., 2016; Moazzezy et al., 2022; Lin et al., 2021
	Defensins	They are a class of AMPs with three disulfide bridges that coordinate α-helix/β-sheet components.	Gram-positive bacteria are resistant to them.	Obaid and Al-Ganahi, 2025; Abdullaeva and Mannanov, 2025
	Cecropins	They are linear amphipathic α-helical AMPs	They are active against Gram-negative bacteria only	Kumari et al., 2025
	Cathelicidins	They are antimicrobial peptides that are tiny, cationic, and vary in amino acid sequence, structure, and size. They are stored in neutrophil and macrophage secretory granules and released extracellularly when leukocytes are activated.	They have a wide range of antimicrobial activity against bacteria, enveloped viruses, and fungi. The cytoplasmic membrane of bacteria is the primary target.	Rebuffat, 2012; Nocek et al., 2012
	Microcins	It's a low-molecular-weight antimicrobial peptide produced as a host defense peptide by Gram-negative enterobacteria. They are smaller than other antimicrobial peptides, measuring about 10 kDa.	They are effective against Gram-negative bacteria such as *E. coli, Salmonella enteritidis*, and *Salmonella typhimurium*. DNA gyrase is targeted to stop DNA replication.	Thangamani et al., 2016
	Auranofin	Because of its capacity to inhibit bacterial protein synthesis, it reduces the generation of important methicillin-resistant Staphylococcus aureus toxins significantly	Multiple biosynthetic processes, including cell wall, DNA, and bacterial protein production, are inhibited	Muñoz-Camargo and Cruz, 2024
	Buforin II	A cationic and linear peptide with 21 amino acids. Crosses the cell membrane without causing it to permeabilize.	DNA replication and protein synthesis are both inhibited	Xie et al., 2011
Combination therapy	Antibiotic–antibiotic	Colistin with tigecycline, aminoglycosides, imipenem, and meropenem	The MIC of this antibiotic combination was reduced by 2.6 to 2.8-fold.	Soudeiha et al., 2017
Phage therapy	OMKO1, wksl3	A novel therapeutic technique in which bacteriophages select MDR bacteria to become more responsive to traditional medicines	The capacity of phages to destroy antibiotic-resistant bacteria, combined with their widespread nature, high specificity, ability to self-replicate at the infection site, and, most crucially, low intrinsic toxicity, qualifies them as a safe and environmentally friendly technology	Bae et al., 2016
Drug, AMP, and essential oil delivery via nanoparticles	Drug, AMP, and essential oil delivery	By altering membrane permeability, cell wall and cytoplasm, as well as causing irreversible damage to bacterial cells, AgNPs of penicillin G, amoxicillin, erythromycin, and vancomycin show enhanced antibacterial and anti-biofilm formation in bacteria such as *Acinetobacter baumannii, Enterococcus faecalis, Klebsiella pneumoniae, Pseudomonas aeruginosa, Staphylococcus*. Biofilm development is also inhibited by Au, Mg, NO, ZnO, CuO, Fe_3_O_4_, and YF NPs	Demonstrated the bactericidal activity of the improvised solution. Because nanoparticles do not enter the bacterial cell, the process by which they kill bacteria is based on direct contact with the cell wall	Chan et al., 2016; Beyth et al., 2015; Franci et al., 2015; Wang L. et al., 2017
Drug delivery vehicles made of liposomes	Liposomes containing drugs	Liposomes are spherical vesicles made up of one or more lipid bilayers surrounding aqueous gaps that are employed as targeted drug delivery devices. Their particle diameters range from 30 nm to several micrometers.	Vancomycin, gentamicin, triclosan, chlorhexidine, benzyl penicillin G, amikacin, tobramycin, meropenem, and other medications are delivered using liposomes such as BBLs (biomineral-binding liposomes), liposome loaded scaffolds (LLSs), and solid-supported liposomes (SSLs). Bacteria such as *E. coli, P. aeruginosa, A. baumannii, S. aureus*, and *S. oralis* produce less biofilm as a result of apoptotic body-like (ABL).	Nag and Awasthi, 2013; Rukavina and Vanić, 2016; Poerio et al., 2017; García et al., 2012
Use of natural chemicals	Flavonoids (isocytisoside eucalyptin)	Flavone, flavanones, flavanols, and anthocyanidins are pigmented chemicals found in plant fruits and flowers.	They are effective against MDR *Pseudomonas aeruginosa, Salmonella typhi, E. coli*, and *Klebsiella pneumoniae*. Increased membrane permeability causes membrane stability to be disrupted.	Chandra et al., 2017
	Alkaloids (berberine)	It's made up of nitrogenous heterocyclic molecules.	*P. aeruginosa, E. coli, S. aureus, S. mutans, M. gypseum, M. canis*, and *T. rubrum* all show wide antibiotic action.	Savoia, 2012
	Coumarins (asphodelin A)	They're aromatic benzopyrones having benzene and alpha pyrone rings bonded together.	They have antibacterial activity against *S. viridians, S. mutans*, etc.	Tillotson and Theriault, 2013
Modification of antimicrobials	Plazomicin (ACHN-490)	Addition of a hydroxyl-aminobutyric acid substituent at position 1 and a hydroxyethyl substituent at position 6 results in a sisomicin derivative.	An aminoglycoside with improved bactericidal action against MDR Gram-negative bacteria and *S. aureus*.	López-Diaz et al., 2017

## 7 Conclusions

Drug resistance is currently a major issue in the field of microbial infection. Bacteria are becoming smarter by demonstrating diverse drug resistance strategies, which not only aid in hostile environments but also produce a smart system for greater nutritional availability for microorganisms. Pathogenic microorganisms are extensively studied in terms of antibiotic resistance. Some prevalent forms of bacterial resistance include enzyme-mediated antibiotic breakdown, quorum sensing, and efflux pump-based antibiotic extrusion. Many studies unraveling the relationship between increased resistance and evolution of ARG show that this link will not only lead to an increased prevalence of current and recurrent infection but probably also increase further emergence of antibiotic resistance. This review emphasized the importance of efflux pumps in drug resistance mechanisms in bacteria and their use to learn about bacterial infections, diseases, and new medicine development. We have also highlighted the genetic and physiological approaches regarding bacterial resistance and conclude that combining novel anti-infective treatments with medication repurposing alone or in conjunction with antimicrobials may be an effective method to battle MDR and persistent pathogenic microorganisms. However, DNA-based diagnostics are hindered due to the lack of understanding of the genetic basis for resistance, as they are mostly detected on the basis of expression-based or physiology-based markers, which are unable to distinguish between antibiotic-resistant cells and alive but non-culturable cells. We believe that marker-based diagnostics will not be successful if the genetic foundation and physiology of antibiotic-resistant cells are well understood. Therefore, a multifaceted one-health approach is needed at the hour for reducing the global burden of antibiotic resistance. Further research should not only focus on exclusively battling antibiotic resistance but also on improving and discovering novel genomic strategies as well as diagnostic tools to detect resistance that may be helpful in preventing resistance from evolving, as well as improving clinical safety and effectiveness.
